# Ginsenoside Rc attenuates DSS-induced ulcerative colitis, intestinal inflammatory, and barrier function by activating the farnesoid X receptor

**DOI:** 10.3389/fphar.2022.1000444

**Published:** 2022-10-28

**Authors:** Kaijia Tang, Danli Kong, Yuan Peng, Jingyi Guo, Yadi Zhong, Haibing Yu, Zhenhua Mai, Yanling Chen, Yingjian Chen, Tianqi Cui, Siwei Duan, Tianyao Li, Naihua Liu, Dong Zhang, Yuanlin Ding, Jiawen Huang

**Affiliations:** ^1^ Science and Technology Innovation Center, Guangzhou University of Chinese Medicine, Guangzhou, China; ^2^ Department of Epidemiology and Medical Statistics, School of Public Health, Guangdong Medical University, Dongguan, China; ^3^ Department of Pharmacy, Affiliated Hospital of North Sichuan Medical College, Nanchong, China; ^4^ Department of Critical Care Medicine, Affiliated Hospital of Guangdong Medical University, Zhanjiang, China; ^5^ The First Clinical Medical College of Guangzhou University of Chinese Medicine, Guangzhou, China; ^6^ The Fourth Clinical Medical College of Guangzhou University of Chinese Medicine, Shenzhen, China

**Keywords:** ulcerative colitis, inflammatory bowel disease, inflammation, ginsenoside Rc, intestinal barriers

## Abstract

**Objectives:** Farnesoid X receptor (FXR) activation is involved in ameliorating inflammatory bowel disease (IBD), such as ulcerative colitis (UC), and inflammatory regulation may be involved in its mechanism. Ginsenoside Rc (Rc) is a major component of *Panax ginseng*, and it plays an excellent role in the anti-inflammatory processes. Our aim is to explore the alleviative effect of Rc on dextran sulfate sodium (DSS)-induced inflammation and deficiencies in barrier function based on FXR signaling.

**Materials and Methods:** *In vitro*, we treated human intestinal epithelial cell lines (LS174T) with LPS to explore the anti-inflammatory effect of Rc supplementation. *In vivo*, a DSS-induced IBD mice model was established, and the changes in inflammatory and barrier function in colons after Rc treatment were measured using the disease activity index (DAI), hematoxylin and eosin (H&E) staining, immunofluorescence, ELISA, and qPCR. Molecular docking analysis, luciferase reporter gene assay, and qPCR were then used to analyze the binding targets of Rc. DSS-induced FXR-knockout (FXR^−/-^) mice were used for further validation.

**Results:** Rc significantly recovered the abnormal levels of inflammation indexes (*TNF-α*, *IL-6*, *IL-1β*, and *NF-KB*) induced by LPS in LS174T. DSS-induced C57BL/6 mice exhibited a significantly decreased body weight and elevated DAI, as well as a decrease in colon weight and length. Increased inflammatory markers (*TNF-α*, *IL-6*, *IL-1β*, *ICAM1*, *NF-KB*, F4/80, and CD11b displayed an increased expression) and damaged barrier function (*Claudin-1*, *occludin,* and *ZO-1* displayed a decreased expression) were observed in DSS-induced C57BL/6 mice. Nevertheless, supplementation with Rc mitigated the increased inflammatory and damaged barrier function associated with DSS. Further evaluation revealed an activation of FXR signaling in Rc-treated LS174T, with *FXR*, *BSEP,* and *SHP* found to be upregulated. Furthermore, molecular docking indicated that there is a clear interaction between Rc and FXR, while Rc activated transcriptional expression of FXR in luciferase reporter gene assay. However, these reversal abilities of Rc were not observed in DSS-induced FXR^−/-^ mice.

**Conclusion:** Our findings suggest that Rc may ameliorate inflammation and barrier function in the intestine, which in turn leads to the attenuation of DSS-induced UC, in which Rc may potentially activate FXR signaling to protect the intestines from DSS-induced injury.

## Introduction

Ulcerative colitis (UC) is a type of inflammatory bowel disease (IBD) that is often characterized by diarrhea, rectal bleeding, abdominal pain, and so on. It is also described as a chronic idiopathic inflammatory disease (Sands, 2004). In the past few decades, high morbidity and disability caused by IBD have resulted in a high cost of treatment and care management, which requires effective prevention strategies (Higgins. et al., 2003; Sterne et al., 2011). Many factors can increase the risk of IBD, such as smoking, urban living, antibiotic exposure, and so on (Piovani et al., 2019). Although the exact pathogenesis of IBD is unknown, the current view holds that it is caused by disrupted homeostasis of the mucosal immune system and impaired intestinal epithelial barriers (Kiesler et al., 2015). Clinically, aminosalicylates, glucocorticoids, and immunosuppressants are commonly used in IBD treatment. However, these drugs can have significant disadvantages, such as unsatisfactory efficacy and many adverse reactions (Lim et al., 2016). Thus, immunomodulators have received increasing attention as potential therapeutics for the treatment of IBD.

Cytokines and inflammatory mediators are the products of IBD inflammatory reactions, which often lead to damage to intestinal epithelial cells, breaking of cell junctions, and finally result in dyshomeostasis of the intestinal flora (Bischoff et al., 2014; Drury et al., 2021). The Farnesoid X receptor (FXR), which is known as a nuclear bile acid receptor, can not only modulate the metabolic balance of bile acid, lipids, and glucose (Sun et al., 2021) but may also be involved in regulating the inflammatory response (Anderson and Gayer, 2021). FXR activation controls the transcriptional induction of the expression of small heterodimer protein (SHP) (Wang et al., 2002), as well as bile salt export pump (BSEP) proteins (Ananthanarayanan et al., 2001). The recruitment of NF-KB can be directly prevented by SHP, which represses several cytokines, including IL-1β and TNF-α (Yang et al., 2016). Both of these can be considered to be FXR targets, and their expression levels have been inversely correlated with inflammation levels (Huang et al., 2018; Cariello et al., 2021). Interestingly, although FXR can modulate inflammatory signaling, acute inflammatory cytokines can also influence FXR expression. For instance, TNFα was able to decrease binding activity with an FXR response element in DNA and resulted in FXR downregulation (Kim et al., 2003). Recently, an increasing number of studies have focused on pharmacologic FXR agonists and FXR has received enthusiastic attention as a developing therapeutic target (Massafra et al., 2018; Badman et al., 2020).

Progressive increases in intestinal permeability have been suggested to be involved in the pathogenesis of IBD (Weber and Turner, 2007; Xavier and Podolsky, 2007). Tight junctions (TJs) are the most important components of intestinal barriers and can be affected by many factors (e.g., immune cells). For example, TJs can be interrupted by TNFα, which downregulates claudin and occludin. This results in increased intestinal permeability (Al-Sadi et al., 2013). It is known that FXR activation can not only inhibit pro-inflammatory cytokine production but can also reduce goblet cell loss and inhibit epithelial permeability (Gadaleta et al., 2011). FXR agonists have been shown to be potential candidates for IBD treatment (Stojancevic et al., 2012).

Ginsenoside Rc (Rc) is a major anti-inflammatory component of *Panax ginseng*, which exhibits anti-oxidative and anti-inflammatory activities through different mechanisms (Yu et al., 2016). A previous study has reported that Rc can reduce inflammatory levels and repair cellular damage in cardiomyocytes (Huang et al., 2021). However, it is unknown whether Rc could ameliorate IBD by reducing the inflammatory response. Furthermore, the association of Rc and FXR has not yet been reported. Here, we hypothesize that Rc may ameliorate IBD symptoms by reducing inflammation and its mechanism may involve the activation of the FXR signaling pathway as an FXR agonist. In this study, we assess the anti-inflammation activity and intestinal damage repair ability of Rc on a dextran sulfate sodium (DSS)-induced IBD mice model. Furthermore, experimental FXR^−/−^and wild-type (WT) mice were used to assess the key role that FXR plays in Rc treatment of IBD. We aimed to explore the anti-inflammatory effects and the functional mechanism of Rc in IBD treatment.

## Materials and methods

### Cell culture

Human intestinal epithelial cell lines LS174T (ATCC) were cultured in fresh DMEM with 10% FBS. Rc (purity >98%, HPLC) was purchased from Shanghai Yuanye Bio-Technology Co., Ltd. (Shanghai, China). HepG2 cells (ATCC) were also maintained in fresh DMEM with 10% FBS. To investigate the cell viability of Rc, LS174T were treated with different concentrations of Rc (0, 6.25, 12.5, 25, 50, 100, 200, and 400 µM). Meanwhile, 24 hours later, cell viability was evaluated *via* the CCK-8 assay. Moreover, to investigate whether Rc can reduce inflammation induced by LPS, cells were pretreated with Rc for 48 h, incubated with LPS (2000 ng/ml) for 24 h, and collected for further analysis.

### Animal experiments

All of the animal experimental studies were approved by the Animal Ethics Committee of Guangzhou University of Chinese Medicine. In total, 50 male C57BL/6 mice (6 weeks old, 20–25 g) were purchased from the Model Animal Research Center of Guangzhou University of Chinese Medicine (Certificate: SCXK 2018-0034; Guangzhou, China). They were housed in an SPF room (25°C, 12 h day/night cycle, free access to chow and water). After 2 weeks, the mice were randomly divided into five groups (Saline, DSS, DSS Rc 5 mg/kg, DSS Rc10 mg/kg, and DSS Rc 20 mg/kg), with 10 mice in each group. The mice were then administered different concentrations of Rc every day throughout the experimental period (11 days). On the third day, the mice were given a 4% DSS solution (w/v) until the end of the experimental period. FXR-knockout (FXR^−/-^) mice were kindly provided by Changhui Liu, and were generated and used as previously purchased from the Jackson Laboratory (Bar Harbor, ME, United States) (Liu et al., 2020). The genotyping for FXR identification is shown in Supplementary Figure S1. Similarly, 18 FXR^−/-^ mice were divided into three groups (Saline, DSS, and DSS Rc 20 mg/kg), and were treated in the same way as those mentioned above. All of the blood and tissues were collected from the mice after anesthesia on the last day.

### Disease activity index

The disease activity index (DAI) was measured following the method of a previous study (DAI score = weight loss (%) + stool consistency + rectal bleeding) (Detel et al., 2012). The weight loss, stool consistency, and rectal bleeding of each mouse were observed daily to evaluate the symptoms of colitis.

### Hematoxylin and eosin staining

The entire colon was weighed and measured at the end of the experiment. Parts of colons were placed in 4% paraformaldehyde and embedded and cut for H&E staining. Images were collected from a light microscope. The remaining parts were stored for further analysis.

### Immunofluorescence

Immunofluorescence was performed according to current protocols (Liu et al., 2018). In brief, colon sections were incubated with F4/80, CD11b, and ZO-1 (Affinity, United States) at 4°C overnight. After washing them three times in PBS, sections were incubated with secondary antibodies (Abclonal, Wuhan, China) for 40 min at room temperature. After washing them five times in PBS, images were observed using a fluorescence microscope (Nikon, Japan). LS174T cells were treated with Rc (25 μM) for 24 h after treatment with adnexal LPS (2000 ng/ml), washed twice with PBS, blocked with 4% paraformaldehyde, incubated with NF-KB primary antibody (Abcam, ab16502) at 4°C overnight, followed by incubation with secondary antibody (Abmart, Alexa Fluor 488) for 1 h, washed three times with PBS, and blocked with DAPI blocker for microscopic observation.

### TNF-α, IL-1β, and IL-6 measurement of serum levels

Serum was collected for TNF-α, IL-1β, and IL-6 measurements. In this experiment, IL-1β and IL-6 were detected by the corresponding ELISA kits from Abclonal (Wuhan, China), while TNF-α was detected by ELISA kits from Ruixinbio (Quanzhou, China), following the manufacturer’s instructions.

### Quantitative PCR

The TRIzol reagent was used for the total mRNA extraction of mouse colon tissue samples. A high-capacity cDNA reverse-transcription kit (Abclonal, Wuhan, China) was used for reverse transcription. Meanwhile, cDNA was subjected to quantitative PCR (qPCR) analysis with the PowerUpTM SYBRTM Green Master Mix (Abclonal, Wuhan, China). The expression levels of all the genes were standardized with *β*-actin and the specific primer sequences are shown in Table 1.

**TABLE 1:** Primers used in qPCR.

Gene name	Forward primer	Reverse primer
*FXR*	GCT​TGA​TGT​GCT​ACA​AAA​GCT​G	CGT​GGT​GAT​GGT​TGA​ATG​TCC
*BSEP*	TCT​GAC​TCA​GTG​ATT​CTT​CGC​A	CCC​ATA​AAC​ATC​AGC​CAG​TTG​T
*SHP*	TGG​GTC​CCA​AGG​AGT​ATG​C	GCT​CCA​AGA​CTT​CAC​ACA​GTG
*IL-6*	TAG​TCC​TTC​CTA​CCC​CAA​TTT​CC	TTG​GTC​CTT​AGC​CAC​TCC​TTC
*ICAM1*	GTG​ATG​CTC​AGG​TAT​CCA​TCC​A	CAC​AGT​TCT​CAA​AGC​ACA​GCG
*COX-2*	TTC​AAC​ACA​CTC​TAT​CAC​TGG​C	AGA​AGC​GTT​TGC​GGT​ACT​CAT
*ZO-1*	GCC​GCT​AAG​AGC​ACA​GCA​A	TCC​CCA​CTC​TGA​AAA​TGA​GGA
*occludin*	TTG​AAA​GTC​CAC​CTC​CTT​ACA​GA	CCG​GAT​AAA​AAG​AGT​ACG​CTG​G
*acludin-1*	GGG​GAC​AAC​ATC​GTG​ACC​G	AGG​AGT​CGA​AGA​CTT​TGC​ACT
h *FXR*	AAC​CAT​ACT​CGC​AAT​ACA​GCA​A	ACA​GCT​CAT​CCC​CTT​TGA​TCC
h *SHP*	GTG​CCC​AGC​ATA​CTC​AAG​AAG	TGG​GGT​CTG​TCT​GGC​AGT​T
h *BSEP*	TTG​GCT​GAT​GTT​TGT​GGG​AAG	CCA​AAA​ATG​AGT​AGC​ACG​CCT
*β-actin*	ATG​ACC​CAA​GCC​GAG​AAG​G	CGG​CCA​AGT​CTT​AGA​GTT​GTT​G
h *β-actin*	GAA​TCA​ATG​CAA​GTT​CGG​TTC​C	TCA​TCT​CCG​CTA​TTA​GCT​CCG

### Molecular docking

The chemical composition collection method was as follows. The ChemDraw 14.0 software was used to draw the 2D structure of small molecule ligands (Rc), and then ChemDraw3D was used to transform the 2D structure into a 3D structure and save it as an MOL2 file. The 3D structure was imported into Discovery Studio. The Prepare Ligands module in Molecules was then used to process small molecules. The energy of small molecules was minimized and a CHARMm force field was used to obtain the prepared small molecules and save them in the MOL2 format. The structural acquisition and preprocessing of protein crystals were as follows. The PDB database (https://www.rcsb.org/) contains data on most of the crystals of biological macromolecules reported to date, including crystal complexes of biological macromolecules and small molecules. This docking study was mainly focused on the FXR protein. Discovery Studio software was used to preprocess the protein. We then deleted the water molecules, hydrogenated and charged them, extracted the original ligand from the structure, and used PyMol to process the protein.

### Luciferase reporter gene assay

HepG2 cells were planted in 12-well plates. After 24 h, cells were transfected with 1 μg hFXR-luc and 1 μg Ramlila luciferase expression vector pCMV-RL-TK (Promega) for 36 h. Here, pCMV-RL-TK was used as an internal control. To measure the effect of Rc on FXR activity, cells were incubated with Rc (0, 6.25, 12, 25 µM). After 24 h, cells were collected for luciferase activity assessment using the Dual Luciferase Reporter Assay System (Promega). Relative luciferase activity was corrected for Renilla luciferase activity of pCMV-RL-TK, and normalized to the activity of the control. The 5’ end of the mouse FXR gene extending from position −1838 bp (relative to the transcription start site) to +47 was cloned into the pGL3-Basic (Promega) luciferase reporter plasmid with the MluI/XhoI sites. The primers that were used for plasmid construction are shown in Table 2.

**TABLE 2:** Primers used in plasmids construction.

FXR Promoter-1838∼+47 luc	5′-ATG​TAT​CTC​TAG​TTG​TCC​TGA​TAT​A-3′
	5′-CGC​TCC​CGG​GCT​CTC​CGC​TAG-3′

### Statistical analysis

All of the results are expressed as the means ± SEMs. The data were evaluated and statistical differences between groups were assessed. For multiple group comparisons, we used Student’s *t*-test and a one-way analysis of variance (ANOVA), followed by a post hoc Tukey test using GraphPad Prism 8.

## Results

### Rc reduces inflammation in LPS-induced LS174T cells

**FIGURE 1:**
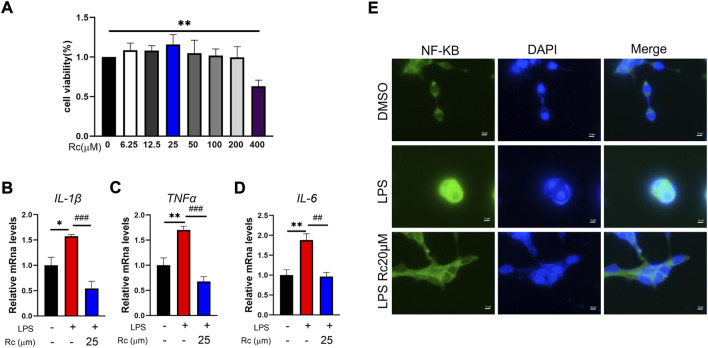
Rc attenuated inflammatory in LPS-induced LS174 cells. Cell viability **(A)** of Rc in LS174 cells (*n* = 3); after incubation with Rc and LPS following the protocol, the cells were collected for qPCR analysis, and the relative mRNA levels of *IL-1β*, *TNF-α* and *IL-6* **(B–D)** were measured (*n* = 4); representative images of immunofluorescence of NF-κB **(E)**. Data are shown as the mean ± SEM. *p < 0.05, **p < 0.01, ***p < 0.001 *vs*. the DMSO group.

We used LPS-induced LS174T cells to establish the IBD cell model *in vitro* to observe the potential therapeutic effect of Rc on UC. In our study, Rc exhibited low cytotoxicity and Rc did not inhibit cell viability up to 400 μM (Figure 1A). According to the result of qPCR analysis, the relative mRNA levels of inflammation, including *IL-1β*, *TNF-α*, and *IL-6* were upregulated in the LPS-induced group. These indices were downregulated with the Rc treatment (Figures 1B–D). Immunofluorescence analysis showed that Rc could inhibit nuclear translocation of NFκB (Figure 1E). These results indicate that Rc may have a potential effect on suppressing inflammation induced by LPS cells.

### Rc treatment reduces colitis symptoms in DSS-induced mice

**FIGURE 2:**
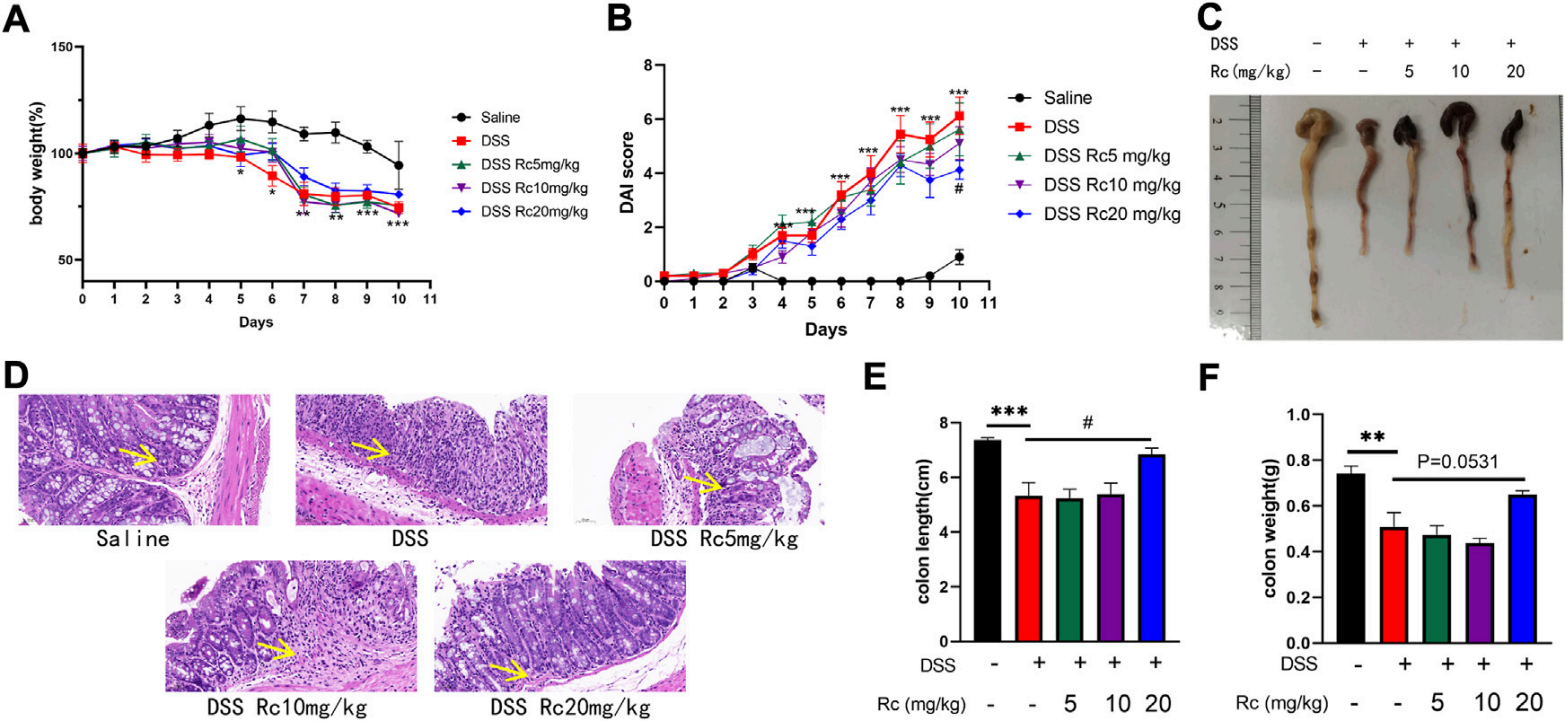
Rc ameliorated DSS-induced UC in mice. Body weight **(A)** changes and disease activity index **(B)** during the whole experiment; macroscopic observation of colon length **(C)**; representative H&E-stained colon sections (magnification: 200 ×) **(D)**; the statistics of colon weight **(E)** and colon length **(F)**. Data are shown as the mean ± SEM (*n* = 10). *p < 0.05, **p < 0.01, ***p < 0.001 *vs*. the saline group; #*p* < 0.05 *vs*. the DSS group.

A DSS-induced mouse model was established to further explore the therapeutic effect of RC on UC. Compared with DSS-treated mice, Rc administration resulted in a dose-dependent weight gain (Figure 2A) and a high dose of Rc slightly reduced DAI scores (Figure 2B). DSS treatment significant shortened colons compared to controls (Figure 2C). H&E staining analysis showed mucosal structural repair, an increased number of crypt structures, and the reduced infiltration of inflammatory cells into the mucosa and submucosa. This indicates that Rc effectively reversed the DSS-induced damage of colon tissues (Figure 2D). Meanwhile, Rc treatment reduced the length of colons and increased the weight of colons in a dose-dependent manner (Figures 2E,F). Taken together, these data suggest that Rc had a very positive impact on the treatment of UC.

### Rc inhibits the inflammatory response in DSS-induced mice

**FIGURE 3:**
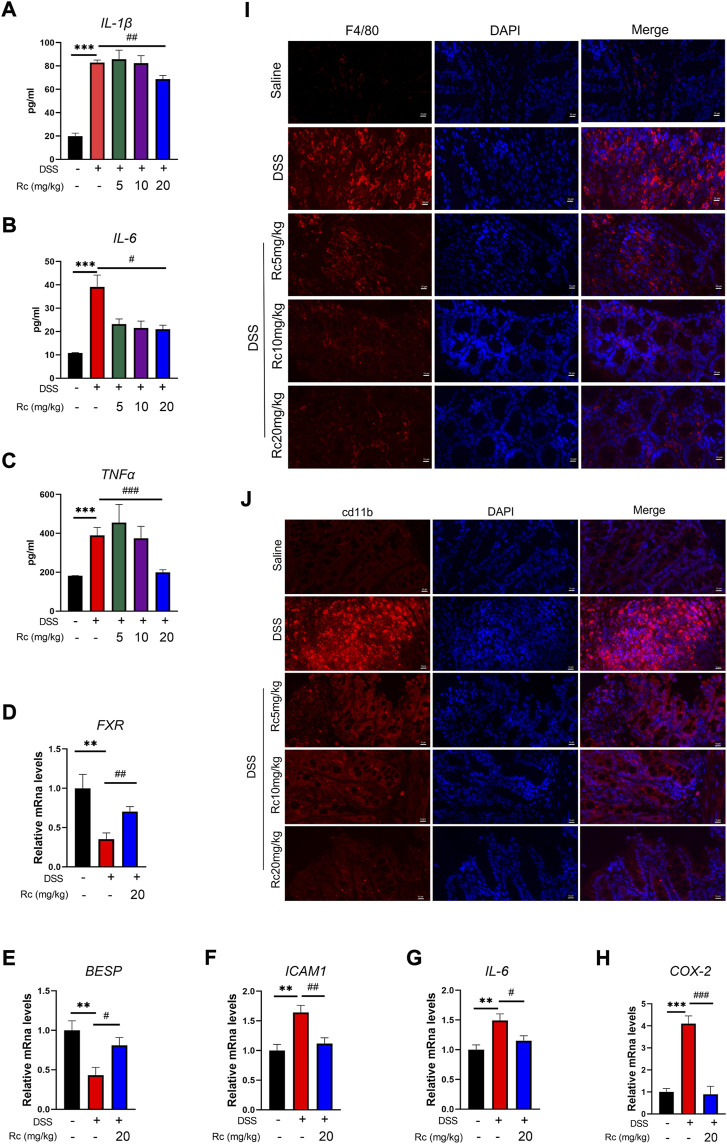
Rc suppressed inflammation by activating FXR in DSS-induced inflamed mouse colons. Serum levels of IL-1β, IL-6 and TNF-α **(A–C)**; the colons were collected for qPCR analysis, and the relative mRNA levels of *FXR* **(D)**, *BSEP* **(E)**, *ICAM* **(F)**, *IL-6* **(G)**, and *COX-2* **(H)** were measured; representative images of immunofluorescence of F4/80 **(I)** and cd11b **(J)** in mouse colon sections (magnification: 200 ×). Data are shown as the mean ± SEM (*n* = 8). *p < 0.05, **p < 0.01, ***p < 0.001 *vs*. the saline group; #*p* < 0.05, ##*p* < 0.01, ###*p* < 0.001 *vs*. the DSS group.

To investigate whether Rc ameliorated the inflammatory response in colitis by activating FXR, we carried out some further studies. First, at the serum level, we found that IL-1β, IL-6, and TNF-α were significantly increased in the DSS-treated model group, whereas high-dose Rc treatment was able to suppress the inflammatory response (Figures 3A–C). The mRNA expression of FXR and its downstream genes were significantly suppressed when compared with the control group, whereas Rc treatment upregulated their expression levels (Figures 3D,E). Similarly, the mRNA levels of DSS-induced pro-inflammatory factors *ICAM1*, *IL-6,* and *COX2* were significantly increased when compared with the normal group, whereas low expressions were found in the Rc-treatment group (Figures 3F–H). Similarly, immunofluorescence analysis showed that Rc reduced the expression of F4/80 and cd11b in a dose-dependent manner (Figures 3I,J). These results indicate that Rc was a potent anti-inflammatory agent, which could ameliorate or even suppress the inflammatory response and alleviate the inflammatory symptoms of UC.

### Rc protects the intestinal mucosa in DSS-induced mice

**FIGURE 4:**
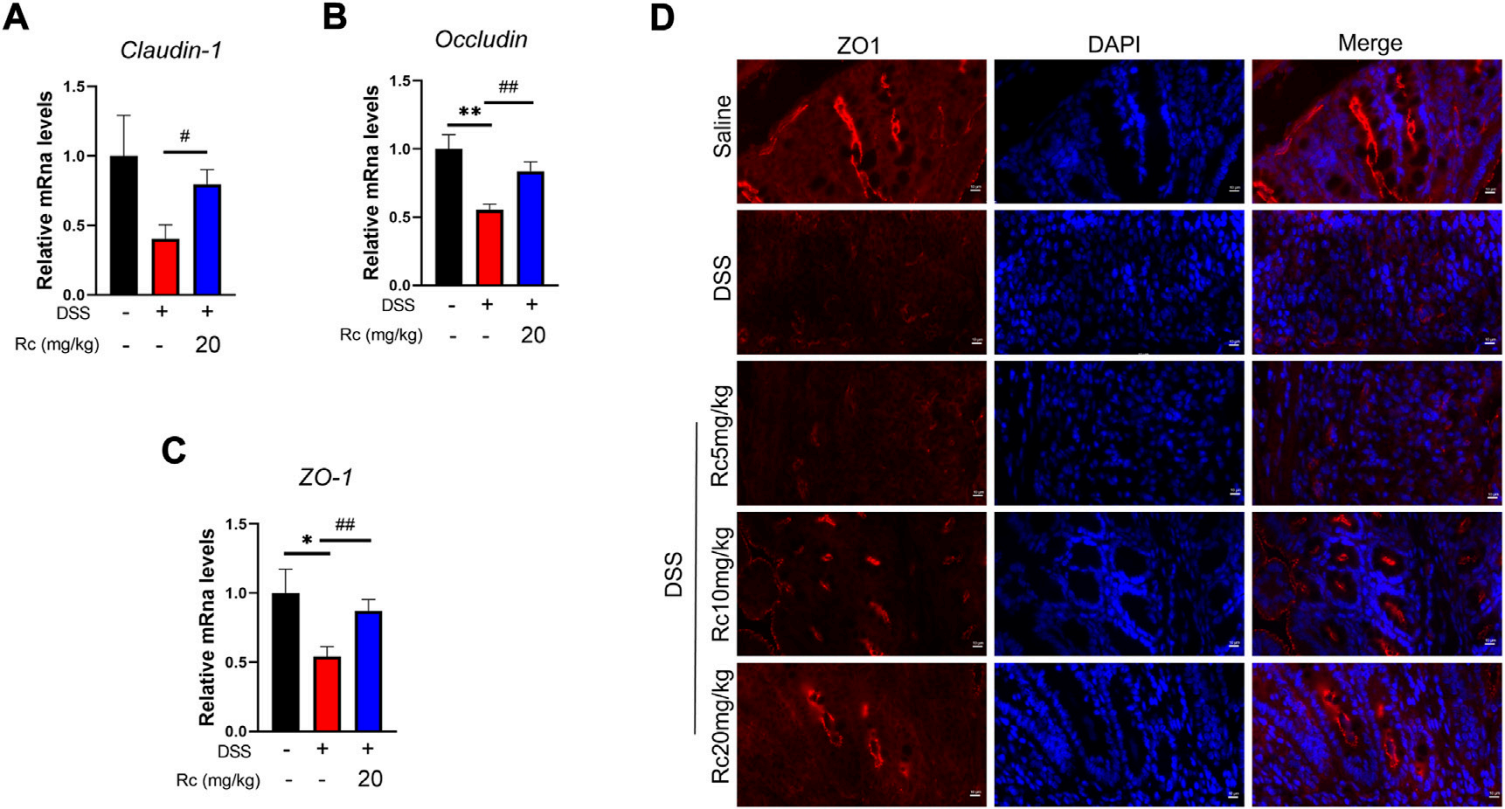
Rc maintains proper tight junctions in DSS-induced mice. The colons were collected for qPCR analysis, and the relative mRNA levels of *claudin-1* **(A)**, *occludin* **(B)**, and *ZO-1* **(C)** were measured; representative images of immunofluorescence of ZO-1 **(D)** in mouse colon sections (magnification: 200 ×). Data are shown as the mean ± SEM (*n* = 6). *p < 0.05, **p < 0.01 *vs*. the saline group; #*p* < 0.05, ##*p* < 0.01 *vs*. the DSS group.

Damage to intestinal barriers is a considerable feature of DSS-induced UC. In our hypothesis we predicted that Rc could not only suppress inflammation but also repair the damage of intestinal barriers induced by DSS. Thus, we focused on TJ molecules. Our results show that Rc upregulated the mRNA expression of *ZO-1*, *claudin-1*, and *occludin* when compared with the DSS group (Figures 4A–C). Meanwhile, the results of immunofluorescence analysis showed that Rc significantly upregulated the expression of *ZO-1* in the inflammatory colon when compared with the DSS group (Figure 4D). Thus, Rc improved the reduction in intestinal permeability brought about by UC.

### Rc activates the FXR signaling pathway

**FIGURE 5:**
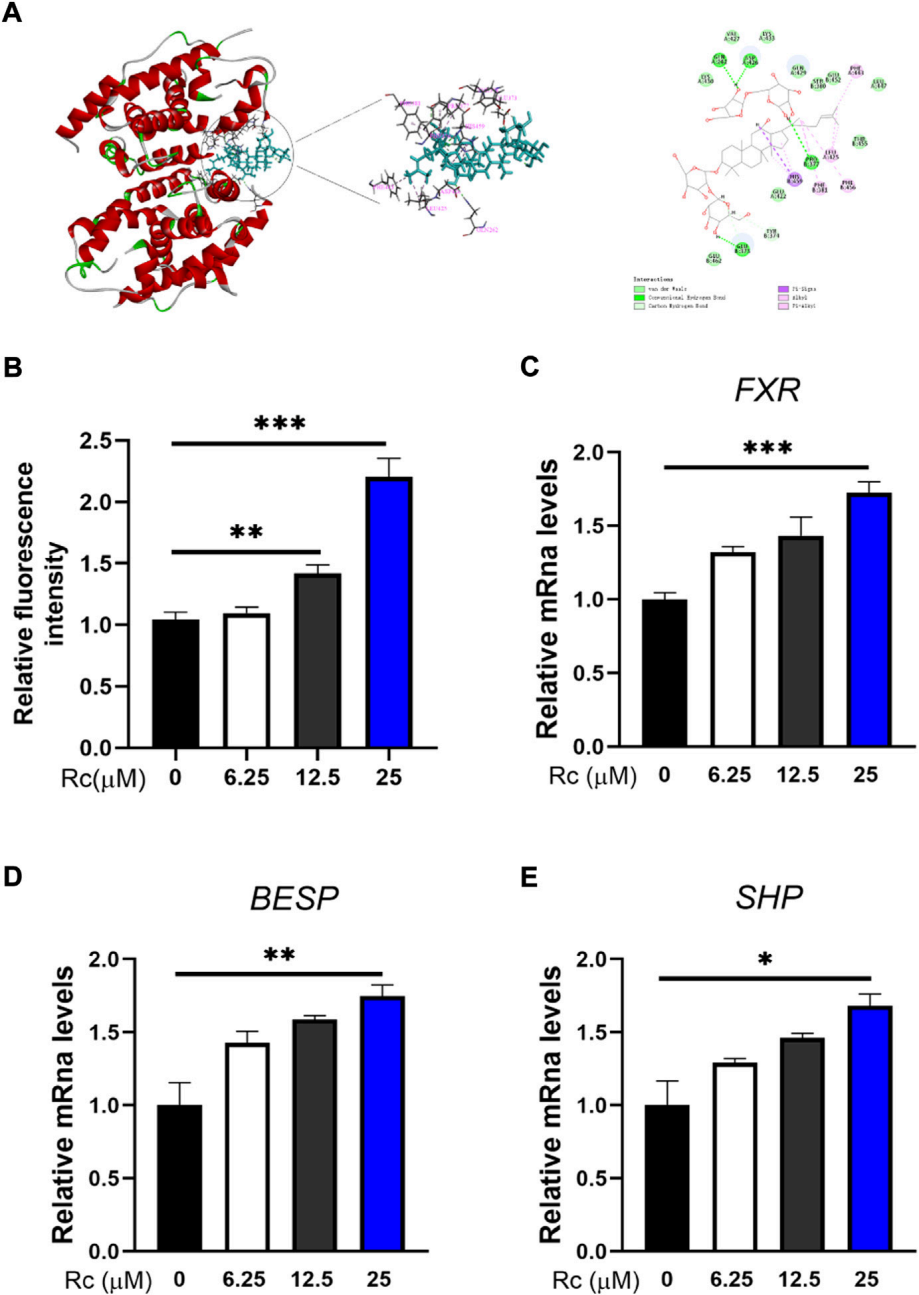
Rc activated FXR signaling pathway in LS174 cells: 3D and 2D molecular docking conformation of Rc with FXR **(A)**; FXR reporter gene activity assay **(B)**; the cells were collected for qPCR analysis, and the relative mRNA levels of *FXR* **(C)**, *BSEP* **(D)**, and *SHP* **(E)** were measured (*n* = 4). Data are shown as the mean ± SEM. *p < 0.05, **p < 0.01, ***p < 0.001 *vs*. the DMSO group.

Molecular docking was conducted to investigate whether Rc can activate FXR. The results show that the inner part of FXR’s structural domain could be bound by Rc based on hydrogen, hydrophobic interaction, and the van der Waals coefficient (Figure 5A). Luciferase reporter gene assay showed significant effect of Rc in activating transcriptional expression of FXR in a dose-dependent manner (Figure 5B). Meanwhile, qPCR results demonstrated that Rc increased the mRNA levels of *FXR*, *BSEP,* and *SHP* in a dose-dependent manner when compared with the control group (Figures 5C–E). These results indicate that Rc could activate the FXR signaling pathway *in vitro*.

### FXR deficiency reduces the protective effect of Rc in DSS-induced FXR−/− mice

**FIGURE 6:**
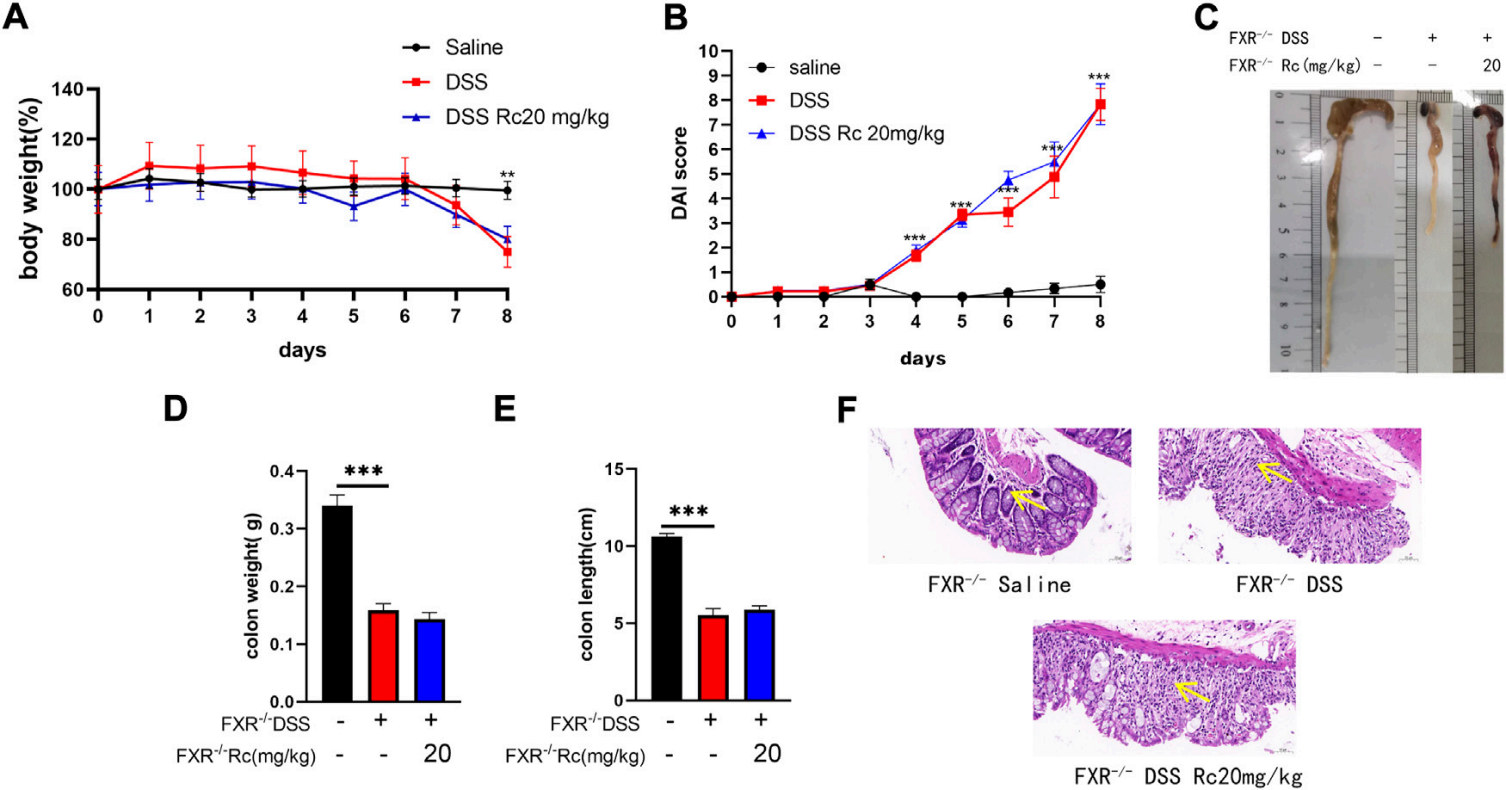
Rc did note ameliorated DSS-induced UC in FXR^−/-^ mice. Body weight **(A)** changes and disease activity index **(B)** during the whole experiment; macroscopic observation of colon length **(C)**; the statistics of colon weight **(D)** and colon length **(E)** representative H&E-stained colon sections (magnification: 200 ×) **(F)**. Data are shown as the mean ± SEM (*n* = 6). **p < 0.01, ***p < 0.001 *vs*. the saline group.

To investigate the effect of FXR on UC, we found that the weight loss and DAI changes caused by DSS were not alleviated after optimal dose treatment with Rc in the case of the knockdown of FXR (Figures 6A,B). Furthermore, the length and weight of the colon were not improved (Figures 6C–E). HE results also showed that the mucosal structure was not repaired, with no change in the mucosa and submucosa in terms of the amount of inflammatory cell infiltration (Figure 6F). This suggests that the absence of FXR renders the treatment of Rc ineffective.

### FXR deficiency deprives Rc of its anti-inflammatory effect in DSS-induced FXR^−/−^ mice

**FIGURE 7:**
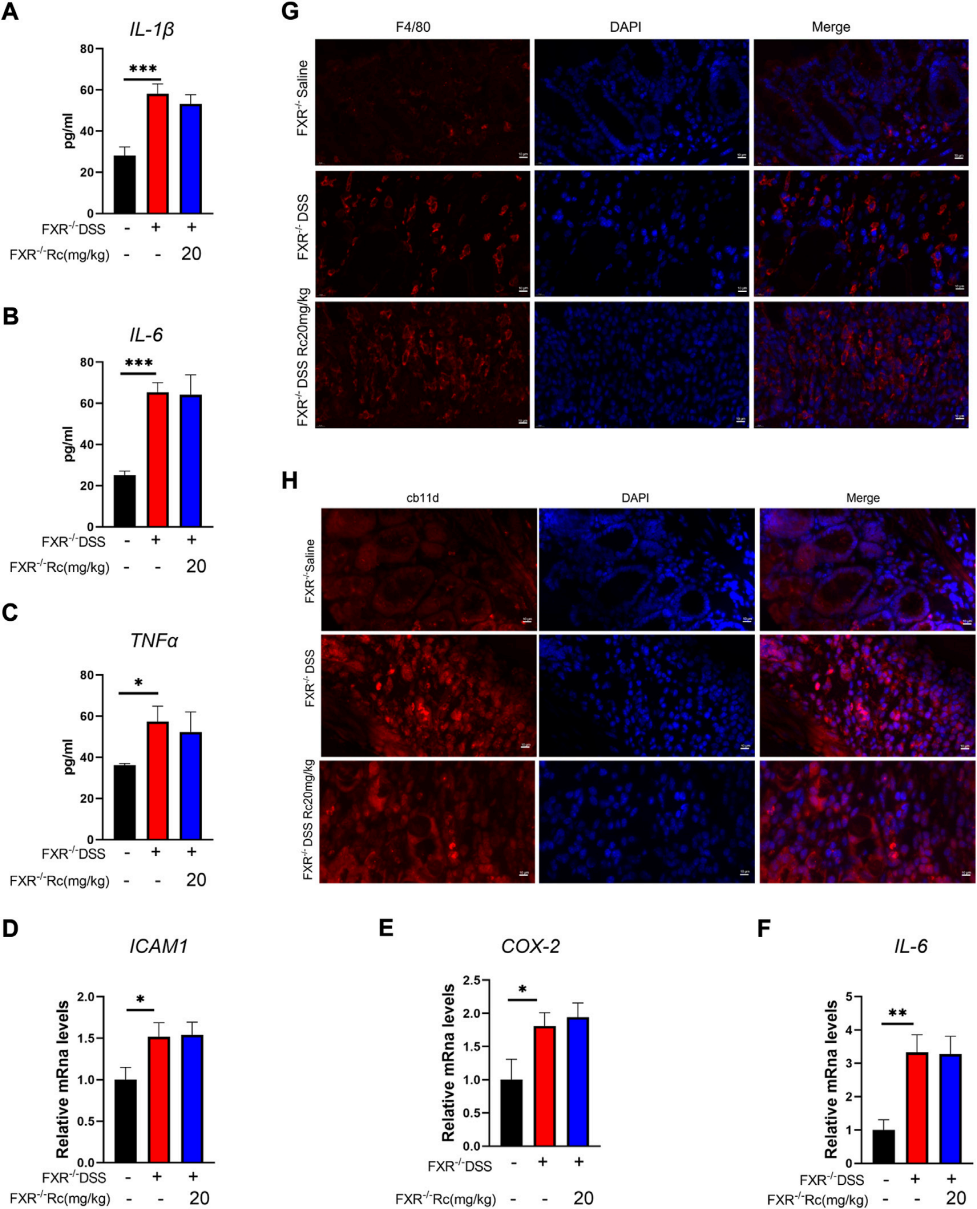
The absence of FXR blocked the effect of Rc on suppressing inflammation in DSS-induced FXR^−/−^ mice. Serum levels of IL-1β, IL-6, and TNF-α **(A–C)**; the colons were collected for qPCR analysis, and the relative mRNA levels of *ICAM* **(D)**, *COX-2* **(E)**, and *IL-6* **(F)** were measured; representative images of immunofluorescence of F4/80 **(G)** and cd11b **(H)** in mouse colon sections (magnification: 200 ×). Data are shown as the mean ± SEM (*n* = 6). *p < 0.05, **p < 0.01, ***p < 0.001 *vs*. the saline group.

Through further studies, we found that under conditions of FXR deficiency, Rc was not able to exert an inhibitory effect on inflammation. At the ELISA level, the expression of IL-1β, IL-6, and TNF-α was not reduced after Rc treatment when compared with the model group (Figures 7A–C). Meanwhile, the mRNA levels of *ICAM1*, *COX-2*, and *IL-6* were also not significantly inhibited (Figures 7D–F). Immunofluorescence results also suggested that Rc failed to produce the inhibition of inflammatory expression levels in the presence of the knockdown of FXR (Figures 7G,H). Therefore, FXR plays an important role in Rc’s therapeutic process in relation to UC inflammation.

### FXR deficiency does not protect intestinal barriers in DSS-induced FXR^−/−^ mice

**FIGURE 8:**
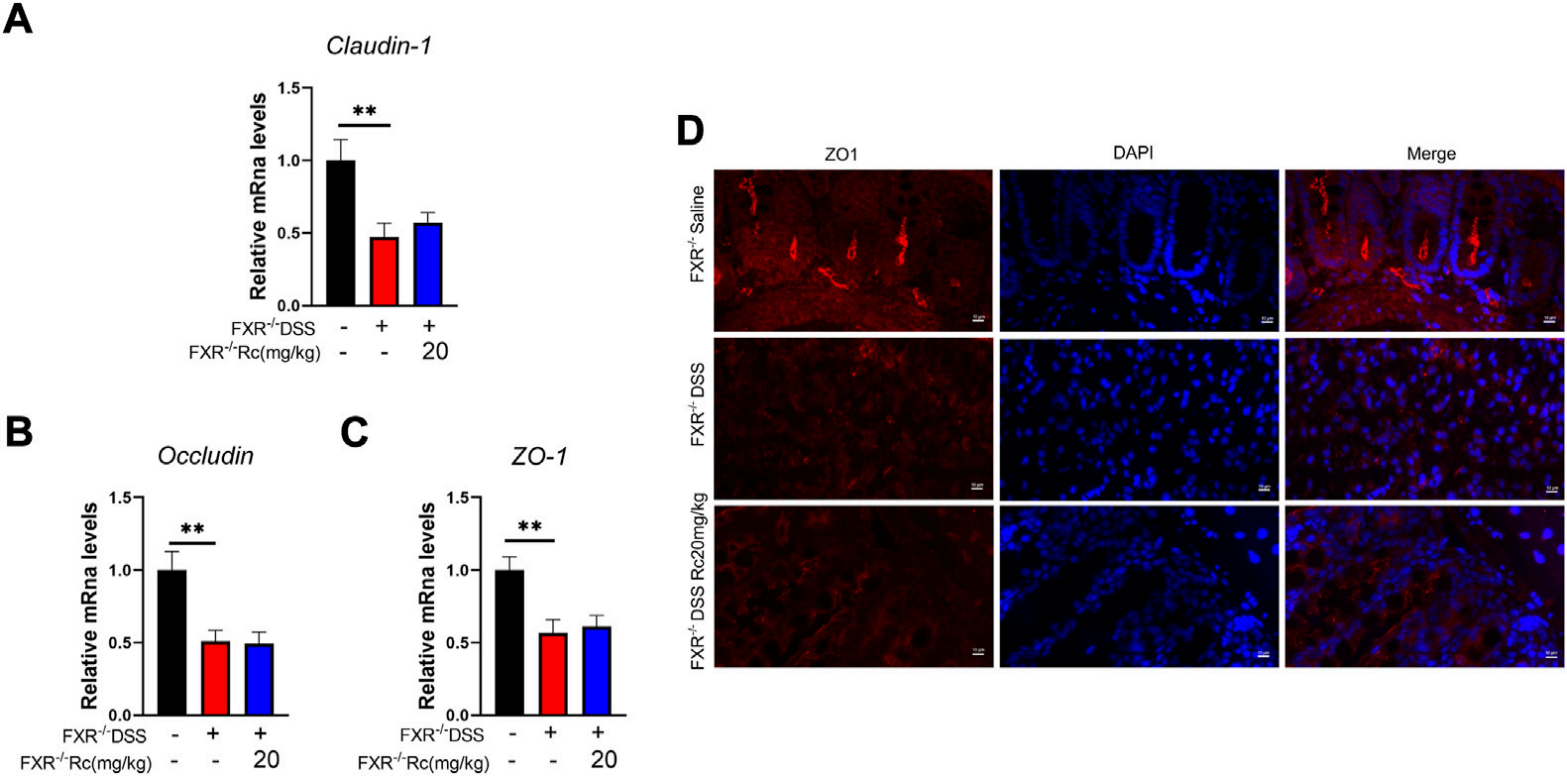
The absence of FXR blocked the effect of Rc maintaining tight junctions in DSS-induced FXR^−/-^ mice. The colons were collected for qPCR analysis, and the relative mRNA levels of *claudin-1* **(A)**, *occludin* **(B)**, and *ZO-1* **(C)** were measured; representative images of immunofluorescence of ZO-1 **(D)** in mouse colon sections (magnification: 200 ×). Data are shown as the mean ± SEM (*n* = 6). **p < 0.01 *vs*. the saline group.

In the absence of FXR, the mRNA expression of *claudin*, *occludin*, and *ZO-1* did not increase after Rc treatment when compared to the model group (Figures 8A–C). Immunofluorescence of ZO-1 also showed no improvement in intestinal permeability (Figure 8D). Overall, Rc treatment could not repair the intestinal mucosal damage caused by DSS after the deletion of FXR. Therefore, FXR may play an active role in repairing the intestinal damage caused by UC and restoring the intestinal mucosa.

## Discussion

Although intestinal barrier disruption is often the final factor causing IBD mortality, intestinal inflammation is often the injury that is observed at the beginning. Consequently, ameliorating inflammation by targeting the FXR signaling pathway represents an attractive concept for combating IBD. Activating FXR might be a therapeutic strategy for treating IBD and its complications. In the present study, we assumed that Rc was an FXR activator, and we verified that Rc ameliorated DSS-induced inflammation and intestinal barrier damage by activating FXR. This therapeutic effect disappeared when FXR was absent (the mechanism diagram is shown in Supplementary Figure S2).

Rc is believed to play an anti-inflammatory role in many disease, such as gastritis, hepatitis, arthritis, and pneumonia (Yu et al., 2016; Lee et al., 2018). Based on the excellent anti-inflammatory ability of Rc, we wondered if Rc could play the same anti-inflammatory role in IBD. Pro-inflammatory cytokines—including IL-1β, IL-6, and TNF-α—have been used to measure inflammation levels in many IBD studies (Hall et al., 2017; Breugelmans et al., 2020). As we expected, Rc reduced the mRNA levels of *IL-1β*, *IL-6*, and *TNF-*α in LPS-induced LS174T *in vitro*, which indicates the potential ability of Rc to attenuate intestinal inflammation. Thus, we then explored its therapeutic effect on an IBD model *in vivo*.

DSS-induced mice are characterized by weight loss, loose stools, diarrhea, and even rectal bleeding. Thus, DSS-induced mice have often been used as an animal model of IBD, including UC and CD (Kim et al., 2012; Liu et al., 2021). Consistently, our study showed symptoms such as decreased body weight, a shortened colon, and increased DAI in DSS-induced mice. In this study, Rc-treated mice exhibited increased colonic weight and length when compared to DSS-induced mice. Furthermore, DSS-induced mice displayed a loss of integrity in their intestinal barriers (e.g., decreased crypt foci and increased inflammatory cell infiltration in the mucosa and submucosa), whereas Rc was able to reverse this damage induced by DSS. Here, we report for the first time that Rc can reverse these UC symptoms that are induced by DSS.

It is clear that FXR activation suppresses the inflammatory response and preserves intestinal barrier integrity in IBD (Ding et al., 2015). We thus assess the role of Rc in ameliorating inflammation and the effects of IBD on intestinal barriers *in vivo*. These pro-inflammatory cytokines can cause intestinal barrier impairment (Nunes et al., 2019). Studies have indicated that IL-1β and TNF-α can cause an obvious increase in intestinal barrier permeability, depending on NF-κB activation (Haines et al., 2016; Zhong et al., 2021). According to our results, Rc suppressed the inflammatory response, which resulted in decreased serum levels of IL-1β, IL-6, and TNF-α. Furthermore, the relative mRNA levels of *ICAM1*, *NF-KB*, *IL-6,* and *COX-2* were increased by DSS but reduced by Rc in our study. These results suggest that Rc may play an anti-inflammatory role by activating the NF-κB pathway in the intestines. Further verification for this hypothesis was carried out with the use of immunofluorescence, and low levels of CD11b and F4/80 inflammatory cells in filtration were observed in the Rc-treatment group. Intestinal macrophages are key immune cells in the maintenance of intestinal immune homeostasis in IBD, and FXR is a modulator of intestinal innate immunity (Vavassori et al., 2009; Dharmasiri et al., 2021). As we expected, the relative mRNA levels of *FXR* and its downstream target *BSEP* were increased with Rc treatment. Mutual crosstalk between FXR and NF-κB might indicate a potential pathway for the anti-inflammatory effect of FXR (Gai et al., 2018). However, more evidence is needed before we can conclude that the activation of FXR directly inhibited the NF-κB pathway. Taken together, our data show that Rc may act as an FXR agonist to reduce intestinal inflammation in DSS-induced mice.

The increasing expression of several types of pro-inflammatory mediators also impaired the intestinal barriers (Al-Sadi et al., 2016). We found that Rc could reduce intestinal inflammation by activating FXR, resulting in NF-κB inhibition. It is known that NF-κB is also a mediator for intestinal barriers, regulating multiple cellular signaling pathways (Cuzzocrea et al., 2000). We then explored its effect on repairing intestinal barriers. As expected, our data showed that Rc improved the relative mRNA levels of *claudin-1*, *occludin*, and *ZO-1*. Further verification was carried out using immunofluorescence, which showed that Rc increased the expression of ZO-1, which indicates that Rc could improve the reduction of intestinal permeability induced by DSS.

FXR is believed to play an anti-inflammatory role and participates in a wide range of diseases of the gastrointestinal tract, such as IBD, colorectal cancer, and type 2 diabetes (Ding et al., 2015). Zhao et al. (2020) found that the downregulation of FXR promoted DSS-induced UC. Meanwhile, Gadaleta et al. (2011) and Feng et al. (2021) found that inflammation could be inhibited by activating FXR. These studies pointed out that the expression of FXR was closely related to DSS-induced UC, which indicates that the regulation of FXR may be useful for ameliorating IBD. Recently, molecular docking has become a commonly used component of the drug discovery toolbox (Luo et al., 2022). Therefore, molecular docking was conducted to explore whether Rc and FXR could interact each other. Remarkably, Rc showed a strong binding affinity to FXR, which indicates that Rc directly regulated the FXR-mediated signaling pathway. The result of the luciferase reporter gene assay further indicates that Rc regulated the transcriptional activity of FXR. Our qPCR results show that the mRNA expressions of *FXR, BSEP*, and *SHP* were increased with Rc treatment. This result is in accordance with those reported for FXR activating its downstream genes BSEP and SHP (Wang et al., 2017; Fu et al., 2022). To our knowledge, this is first time that Rc has been reported as an FXR regulator.

Previously, Verbeke et al. (2015) demonstrated a crucial protective role for FXR in cholestatic rats, which meant that FXR agonists could prevent gut barrier dysfunction, observing upregulated claudin-1 and occludin. Novel drugs have been reported that could improve intestinal barrier function by increasing FXR signaling, which resulted in the alleviation of colitis (Song et al., 2019; Dong et al., 2021). Knowing that the effect of Rc on anti-inflammatory and intestinal barrier repair may occur due to FXR activation, we aimed to explore whether FXR activation is essential for Rc to exert its therapeutic effect. Although FXR was lowly expressed in the intestine in DSS-induced mice, FXR-knockout (FXR^−/-^) mice did not present high DAI scores, whereas DSS-induced FXR^−/-^ mice showed higher DAI scores, lower colon weights, and shorter colon lengths, as well as rising inflammation levels and damaged intestinal barriers. This indicates that DSS also led to UC in FXR^−/-^ mice. However, Rc did not exhibit its therapeutic effect on UC in FXR^−/-^ mice, with no change in the indexes of inflammation and intestinal barriers. Despite the multiple mechanisms behind UC treatment, here the therapeutic effect of Rc on UC appeared to only be achieved by activating FXR. Our findings explain the protective effect of Rc on UC, and they provide evidence that FXR constitutes a valid therapeutic target activated by Rc in the treatment of IBD.

## Conclusion

Our study suggests that Rc may ameliorate inflammatory and barrier function in the intestines. This leads to the attenuation of DSS-induced UC, in which Rc may potentially activate FXR signaling to protect the intestines from DSS-induced injury.

## Data Availability

The original contributions presented in the study are included in the article/Supplementary Material; further inquiries can be directed to the corresponding authors.
