# Supplementary material for: Ginsenoside Rc attenuates DSS-induced ulcerative colitis, intestinal inflammatory, and barrier function by activating the farnesoid X receptor
Source: Front Pharmacol. 2022 Oct 28;13:1000444. doi: 10.3389/fphar.2022.1000444 (PMC9649634; doi:10.3389/fphar.2022.1000444)
Supplement: Supplementary file 1 [file DataSheet1.PDF]

**Supplementary Figures**

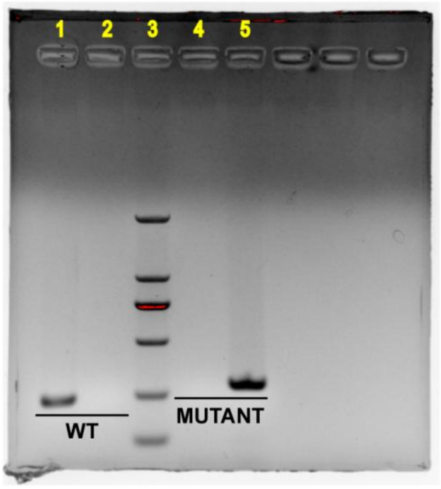


**Supplementary Figure 1** Genotyping of WT/FXR KO mice (1&4-WT mice, 2&5-FXR KO mice, 3-Marker).

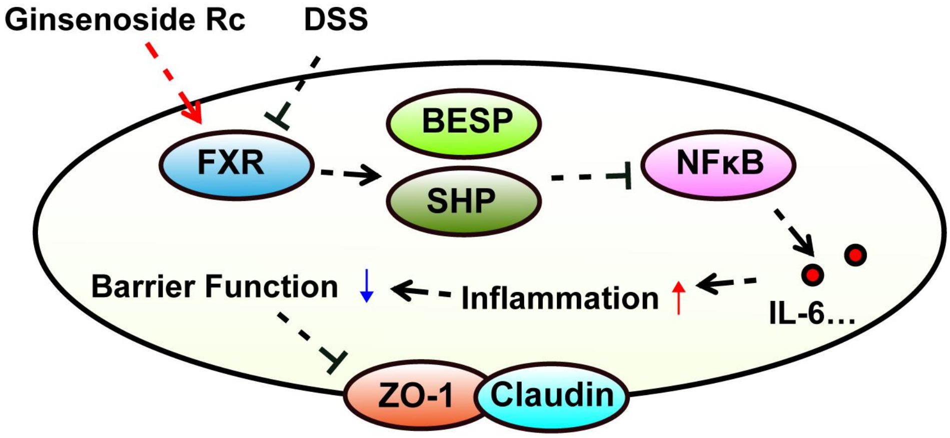


**Supplementary Figure 2** Ginsenoside Rc Attenuates DSS-induced Ulcerative Colitis, Intestinal Inflammatory and Barrier Function by Activating Farnesoid X Receptor.
